# Clinical and occupational risk factors for coronavirus disease 2019 (COVID-19) in healthcare personnel

**DOI:** 10.1017/ash.2022.250

**Published:** 2022-07-18

**Authors:** Jennie H. Kwon, Philip J. Budge, Caroline A. O’Neil, Kate Peacock, Eva M. Aagaard, Victoria J. Fraser, Margaret A. Olsen, Hilary Babcock, for the CDC Prevention Epicenters Program

**Affiliations:** 1 Division of Infectious Diseases, Washington University School of Medicine, St Louis, Missouri; 2 Division of General Medicine and Occupational Health, Washington University School of Medicine, St Louis, Missouri

## Abstract

**Objective:**

To identify characteristics associated with positive severe acute respiratory coronavirus virus 2 (SARS-CoV-2) polymerase chain reaction (PCR) tests in healthcare personnel.

**Design:**

Retrospective cohort study.

**Setting:**

A multihospital healthcare system.

**Participants:**

Employees who reported SARS-CoV-2 exposures and/or symptoms of coronavirus disease 2019 (COVID-19) between March 30, 2020, and September 20, 2020, and were subsequently referred for SARS-CoV-2 PCR testing.

**Methods:**

Data from exposure and/or symptom reports were linked to the corresponding SARS-CoV-2 PCR test result. Employee demographic characteristics, occupational characteristics, SARS-CoV-2 exposure history, and symptoms were evaluated as potential risk factors for having a positive SARS-CoV-2 PCR test.

**Results:**

Among 6,289 employees who received SARS-CoV-2 PCR testing, 873 (14%) had a positive test. Independent risk factors for a positive PCR included: working in a patient care area (relative risk [RR], 1.82; 95% confidence interval [CI], 1.37–2.40), having a known SARS-CoV-2 exposure (RR, 1.20; 95% CI, 1.04–1.37), reporting a community versus an occupational exposure (RR, 1.87; 95% CI, 1.49–2.34), and having an infected household contact (RR, 2.47; 95% CI, 2.11–2.89). Nearly all HCP (99%) reported symptoms. Symptoms associated with a positive PCR in a multivariable analysis included loss of sense of smell (RR, 2.60; 95% CI, 2.09–3.24) or taste (RR, 1.75; 95% CI, 1.40–2.20), cough (RR, 1.95; 95% CI, 1.40–2.20), fever, and muscle aches.

**Conclusions:**

In this cohort of >6,000 healthcare system and academic medical center employees early in the pandemic, community exposures, and particularly household exposures, were associated with greater risk of SARS-CoV-2 infection than occupational exposures. This work highlights the importance of COVID-19 prevention in the community and in healthcare settings to prevent COVID-19.

Healthcare personnel (HCP) are on the front lines of the coronavirus disease 2019 (COVID-19) pandemic. Many HCP have been infected with severe acute respiratory syndrome coronavirus 2 (SARS-CoV-2), the virus that causes COVID-19.^1^ Although many HCP are concerned about occupational exposure to SARS-CoV-2, exposures at home and in the community play an important role in the development of COVID-19 in HCP.^2^ More data concerning occupational and nonoccupational risk factors for COVID-19 are needed to protect HCP from infection.^3^

At the start of the COVID-19 pandemic, our large, regional, academic medical center and its affiliated multihospital healthcare system created a COVID-19 occupational health call center to expand occupational health capacity and implement public health recommendations for testing and work exclusion based on employee symptoms and/or exposures. Employees, including individuals with and without direct patient contact, who had symptoms concerning for COVID-19 or SARS-CoV-2 exposures were instructed to contact the call center. Trained call-center operators (primarily nurses and nurse practitioners) collected demographic, occupational, exposure, and symptom information from callers and followed a script and decision algorithm to refer employees to obtain nasopharyngeal SARS-CoV-2 polymerase chain reaction (PCR) testing when indicated. Details from call-center encounters and associated PCR test results were documented in the electronic medical record. The objective of this study was to better define clinical and occupational risk factors for COVID-19 in HCP utilizing this data set.

## Methods

This study took place at Washington University School of Medicine (WU) and the BJC HealthCare System (BJC), which together have >40,000 employees. BJC comprises 15 hospitals, including a large urban academic medical center and a pediatric hospital affiliated with Washington University, 8 suburban community hospitals, 2 rural hospitals, 4 non–acute-care facilities, and numerous outpatient sites in Missouri and Illinois. The study protocol was reviewed and approved by the WU Human Research Protection Office with a waiver of informed consent.

**Fig. 1.**
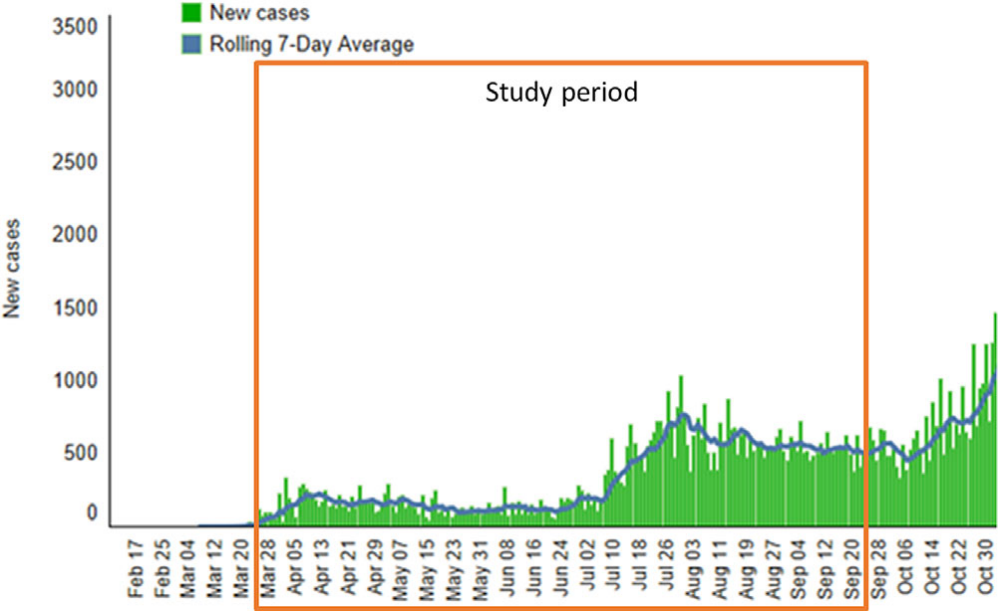
Regional daily SARS-CoV-2 infection rate during the study period. Data from the BJC analytics team, per an internal dashboard, accessed August 24, 2021.

Data from employees aged ≥18 years who contacted the call center between March 30, 2020, and September 20, 2020, were abstracted from the electronic medical record. During this period, the number of new COVID-19 cases in the region was relatively small until the beginning of June, when cases increased substantially. Case numbers then remained stable through the end of the study period. Data from the BJC analytics team from an internal dashboard are shown in Figure 1.

For employees who were referred for SARS-CoV-2 PCR testing, the PCR test results were linked to the associated call-center encounter. Call-center encounters were excluded if they had no associated SARS-CoV-2 PCR test, a date-entry error, or if the caller was aged >90 years. Examples of date-entry errors included reported exposure dates that occurred after the date of the call encounter or reported symptom onset that occurred >14 days prior or >7 days after the encounter date. Repeat calls from a single individual made within 3 days were treated as a single encounter because employees could call repeatedly for the same event. For employees who made multiple calls >3 days apart, 1 call per person was randomly selected for inclusion in the study. PCR tests ordered >7 days after the selected call-center encounter were also excluded because they could not be definitively linked with that encounter.

Data collected by call-center operators and used in the analysis included employee demographics, job role, facility (eg, hospital, non–acute-care facility, or nonhospital setting, including outpatient facilities and the medical school), primary work location (working on site versus working from home), SARS-CoV-2 exposure history, and symptoms. Individuals employed by BJC, WU employees who stated that they entered patient care areas, and students on clinical rotations were classified as healthcare personnel (HCP). Washington University employees who stated that they did not enter patient care areas or only entered patient care areas for research purposes and students not on clinical rotations were classified as non-HCP. Reported SARS-CoV-2 exposures were classified as occupational or community exposures based on the employee’s description of the exposure, when available, and the call-center operator’s assessment. Missing variables were classified as “not documented.” All SARS-CoV-2 PCR testing was conducted by the BJH microbiology laboratory.

After collecting details about reported SARS-CoV-2 exposures, call-center operators used a standardized decision algorithm based on CDC guidance^4^ to designate exposures as high, medium, or low SARS-CoV-2 risk (Supplementary Table 1). Ongoing household contact with someone known or suspected to have COVID-19 was treated as a separate risk category.

The χ^2^ and Student *t* tests were used to examine associations between employee characteristics, SARS-CoV-2 exposure history, and symptoms with positive versus negative SARS-CoV-2 PCR test results. The Fisher exact test was used for categorical variables with small sample sizes. Unadjusted relative risks and 95% confidence intervals were estimated using generalized linear models with log link and binomial distribution. Stratified analyses were performed to examine the association between the exposure variables and PCR test results separately for employees with occupational exposures and community exposures. Symptoms associated with a positive SARS-CoV-2 PCR test were identified using a generalized linear model with log link and Poisson distribution, with calculation of robust standard errors. Variables with *P* < .10 in bivariate analysis were included in the initial full model and were removed in a backward stepwise manner with *P <* .05 as the threshold for retention. All statistical analyses were performed in SAS version 9.4 software (SAS Institute, Cary, NC), and *P* < .05 was considered statistically significant.

## Results

**Fig. 2.**
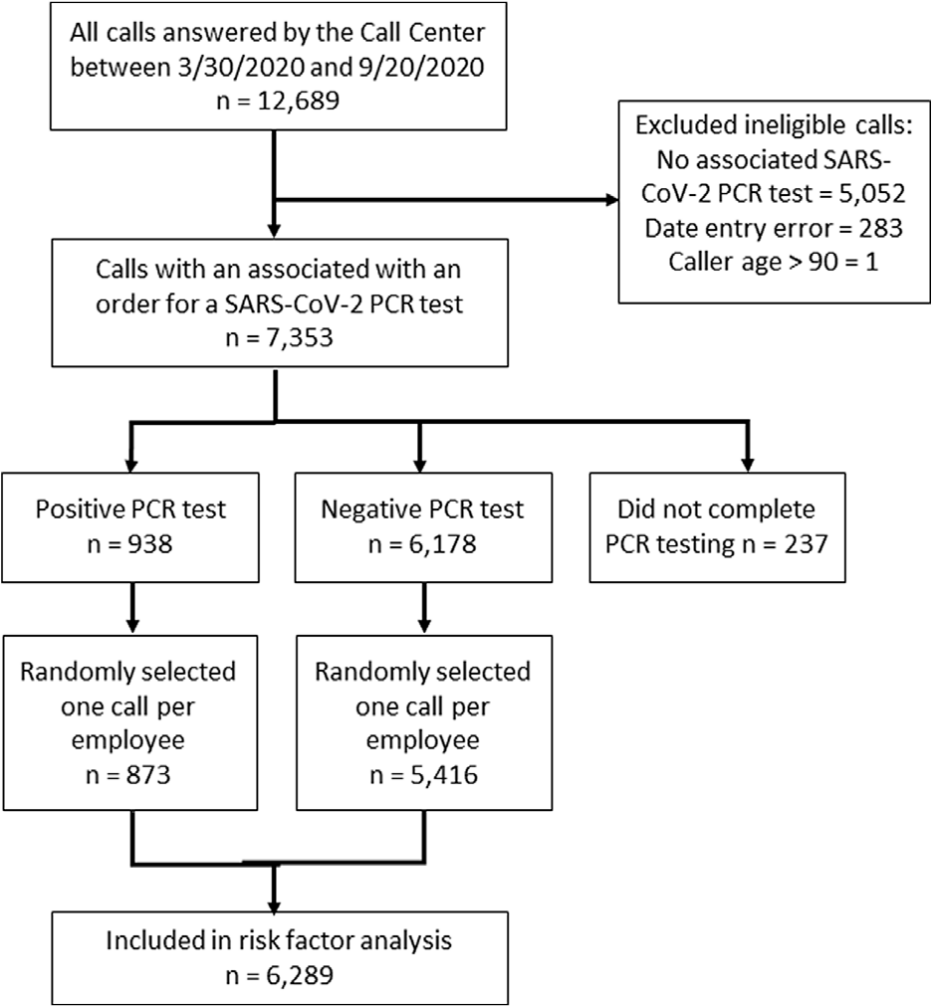
Cohort selection flowchart.

In total, 12,689 calls made by 10,007 employees were answered by the call center during the study period. We excluded 5,336 calls: 5,052 (40%) for not having an associated SARS-CoV-2 PCR test (testing was not indicated for asymptomatic individuals with no or low-risk exposures), 283 (3%) for date entry errors, and 1 (0%) for caller age >90 years (Fig. 2). Among the 7,353 remaining calls, 5,547 employees made 1 call, 668 made 2 calls, and 74 made ≥3 calls. After 1 call was randomly selected for employees with multiple calls, 6,289 calls were included in the analysis: 873 calls (14%) associated with a positive PCR test and 5,416 calls (86%) associated with a negative PCR test (Fig. 2).

Table 1 shows the characteristics and SARS-CoV-2 exposure history for employees with positive versus negative PCR tests. For both groups, the median age was 36 years and 81% were female. The largest group of employees (36%) worked at the urban academic hospital, and fewer worked at suburban or rural community hospitals, the urban pediatric hospital, non–acute-care facilities, or in a nonhospital setting. Compared to employees at the urban academic hospital, employees at the suburban community hospitals had a 1.6-fold increased risk of a positive PCR (relative risk [RR], 1.62; 95% confidence interval [CI], 1.40–1.88), and employees working in a nonhospital setting had lower risk (RR, 0.73; 95% CI, 0.59–0.92). We detected no risk difference for employees working at non–acute-care facilities or rural hospitals (Table 1). HCP working in a patient care area had greater risk for a positive PCR than non-HCP (RR, 1.82; 95% CI, 1.37–2.40) (Table 1). However, compared to employees who worked from home, employees working on site did not have increased risk of a positive PCR test (RR, 1.12; 95% CI, 0.89–1.41).

**Table 1.** Characteristics of Healthcare System and Academic Medical Center Employees Who Contacted the COVID-19 Occupational Health Call Center to Report a SARS-CoV-2 Exposure and Were Referred for SARS-CoV-2 PCR Testing

Variable	All Employees (N=6,289), No. (%)	Employees With a Positive PCR Test (N=873), No. (%)^a^	Employees With a Negative PCR Test (N=5,416), No. (%)^a^	RR (95% CI)	*P* Value
**Demographic characteristics**					
Age, median y (IQR)	36.0 (29.0–48.0)	36.0 (28.0–49.0)	36.0 (29.0–48.0)	1.00 (0.99–1.01)	.79
Sex, female	5,099 (81.1)	711 (81.4)	4,388 (81.0)	1.03 (0.88–1.20)	.73
Not documented	3 (0.1)	1 (0.1)	2 (0.0)	2.46 (0.49–12.25)	.27
**Occupational characteristics**					
**Employment status^b^**					
Employee	1,642 (26.1)	260 (29.8)	1,382 (25.5)	Reference	
Student	39 (0.6)	2 (0.2)	37 (0.7)	0.32 (0.08–1.26)	.10
Not documented	4 (0.1)	1 (0.1)	3 (0.1)	1.59 (0.29–8.65)	.60
**Type of facility where employed^c^**					
Urban academic hospital	2,256 (35.8)	277 (31.7)	1,979 (36.5)	Reference	
Urban pediatric hospital	626 (10.0)	71 (8.1)	555 (10.2)	0.92 (0.72–1.18)	.53
Suburban community hospital	1,553 (24.7)	309 (35.4)	1,244 (23.0)	1.62 (1.40–1.88)	<.001
Rural hospital	193 (3.1)	26 (3.0)	167 (3.1)	1.10 (0.75–1.60)	.63
Non–acute-care facility	78 (1.2)	11 (1.3)	67 (1.2)	1.15 (0.66–2.01)	.63
Nonhospital setting	943 (15.0)	85 (9.7)	858 (15.8)	0.73 (0.58–0.92)	.009
Not documented	601 (9.6)	92 (10.5)	509 (9.4)	1.25 (1.00–1.55)	.047
**Job role**					
Non-HCP^d^	601 (9.6)	48 (5.5)	553 (10.2)	Reference	
HCP^e^	5,688 (90.4)	825 (94.5)	4,863 (89.8)	1.82 (1.37–2.40)	<.001
**Work location**					
Working from home	552 (8.8)	69 (7.9)	483 (8.9)	Reference	
Working on campus	5,639 (89.7)	789 (90.4)	4,850 (89.5)	1.12 (0.89–1.41)	.34
Not documented	98 (1.5)	15 (1.7)	83 (1.5)	1.22 (0.73–2.05)	.44
**Exposure history**					
SARS-CoV-2 exposure status					
No known exposure	3,288 (52.3)	419 (48.0)	2,869 (53.0)	Reference	
Known exposure	2,064 (32.8)	315 (36.1)	1,749 (32.3)	1.20 (1.04–1.37)	.009
Not documented	937 (14.9)	139 (15.9)	798 (14.7)	1.16 (0.97–1.39)	.09
**Type of exposure^f^**					
Occupational exposure	1,750 (27.8)	236 (27.0)	1,514 (27.9)	Reference	
Community exposure	314 (5.0)	79 (9.0)	235 (4.3)	1.87 (1.49–2.34)	<.001
**Operator exposure risk assessment^g^**					
Low	3,249 (51.7)	369 (42.3)	2,880 (53.2)	Reference	
Medium	1,306 (20.8)	165 (18.9)	1,141 (21.1)	1.11 (0.94–1.32)	.23
High	722 (11.5)	114 (13.1)	608 (11.2)	1.39 (1.14–1.69)	.001
Household contact	632 (10.1)	177 (20.3)	455 (8.4)	2.47 (2.11–2.89)	<.001
Not documented	380 (6.0)	48 (5.5)	332 (6.1)	1.11 (0.84–1.47)	.46

Note. HCP, healthcare personnel; IQR, interquartile range; PCR, polymerase chain reaction; PPE, personal protective equipment.

a All percentages are column percentages.

b Because this question was added to the call center script on August 12, 2020, data were only available for 1,907 employees, 1,614 with a negative PCR test and 293 with a positive PCR test.

c Results for facility type do not include respondents who reported that they were students.

d Included employees who did not work in a patient care area.

e Included employees who worked in a patient care area in any capacity.

f Type of exposure only determined for the 2,064 employees who reported a known exposure.

g Exposure risk assessment was determined by call-center operators using a standardized decision algorithm incorporating details about the PPE worn by the employees and the patient and coworker to whom they were exposed, as well as type of care that was provided to the patient. Call Center operators may have determined that there was ongoing household contact for some employees who had initially responded “no” to the question about household contact based on details provided by the employees.

When asked about potential SARS-CoV-2 exposures, 33% of employees reported a known, specific exposure event, and 52% had no known exposure. Employees with a known exposure had greater risk of a positive PCR test than those with no known exposure (RR, 1.20; 95% CI, 1.04–1.37) (Table 1). Among the 2,064 employees reporting a known exposure, 1,750 (85%) reported an exposure that occurred at work (occupational exposure), and only 314 (15%) reported an exposure that occurred outside work (community exposure). Employees who reported community exposures had a greater risk of a positive PCR test than those who reported occupational exposures (RR, 1.87; 95% CI, 1.49–2.34). Stratified analyses did not identify many differences in the characteristics associated with positive SARS-CoV-2 PCR tests among employees who reported occupational versus community exposures (Table 2). However, among employees with occupational exposures, those working at suburban community hospitals had greater risk of a positive PCR test than those working at the urban academic hospital (RR, 2.10; 95% CI, 1.56–2.82). This association was not evident among employees with community exposures (RR, 1.11; 95% CI, 0.69–1.79) (Table 2).

**Table 2.** Characteristics of Healthcare System and Academic Medical Center With Occupational and Community SARS-CoV-2 Exposures, by SARS-CoV-2 PCR Test Status

	Occupational Exposures					Community Exposures				
	All Employees (N=1,750), No. (%)	Employees With a Positive PCR Test (N=236), No. (%)	Employees With a Negative PCR Test (N=1,514), No. (%)	RR (95% CI)	*P* Value	All Employees (N=314), No. (%)	Employees With a Positive PCR Test (N=79), No. (%)	Employees With a Negative PCR Test (N=235), No. (%)	RR (95% CI)	*P* Value
**Demographic characteristics**										
Age, median y (IQR)	36 (29–47)	36 (28–47)	36 (29–47)	1.00 (0.98–1.01)	.90	36 (28–48)	37 (28–50)	36 (27–47)	1.00 (0.99–1.02)	.68
Sex, female	1,441 (82.3)	195 (82.6)	1,246 (82.3)	1.02 (0.74–1.39)	.92	265 (84.4)	63 (79.8)	202 (86.0)	0.73 (0.46–1.15)	.17
Not documented	1 (0.1)	0 (0.0)	1 (0.1)			0 (0.0)	0 (0.0)	0 (0.0)		
**Occupational characteristics**										
**Type of facility where employed**										
Urban academic hospital	596 (34.1)	57 (24.1)	539 (35.6)	Reference		102 (32.5)	27 (34.2)	75 (31.9)	Reference	
Urban pediatric hospital	136 (7.8)	9 (3.8)	127 (8.4)	0.69 (0.35–1.36)	.29	39 (12.4)	10 (12.7)	29 (12.3)	0.97 (0.52–1.81)	.92
Suburban community hospital	568 (32.5)	114 (48.3)	454 (30.0)	2.10 (1.56–2.82)	<.001	75 (23.9)	22 (27.8)	53 (22.5)	1.11 (0.69–1.79)	.67
Rural hospital	42 (2.4)	5 (2.1)	37 (2.4)	1.24 (0.53–2.94)	.62	13 (4.1)	5 (6.3)	8 (3.4)	1.45 (0.68–3.11)	.34
Non–acute-care facilitiy	29 (1.7)	3 (1.3)	26 (1.7)	1.08 (0.36–3.25)	.89	6 (1.9)	1 (1.3)	5 (2.1)	0.63 (0.10, 3.88)	.62
Nonhospital setting	103 (5.9)	6 (2.5)	97 (6.4)	0.61 (0.27–1.38)	.23	64 (20.4)	13 (16.5)	51 (21.7)	0.77 (0.43–1.37)	.37
Not documented	276 (15.8)	42 (17.8)	234 (15.5)	1.59 (1.10–2.31)	.014	8 (2.5)	0 (0.0)	8 (3.4)	Undefined	
**Job role**										
Non-HCP^a^	31 (1.8)	4 (1.7)	27 (1.8)	Reference		43 (13.7)	7 (8.9)	36 (15.3)	Reference	
HCP^b^	1,719 (98.2)	232 (98.3)	1,487 (98.2)	1.05 (0.42–2.63)	.92	271 (86.3)	72 (91.1)	199 (84.7)	1.63 (0.81–3.31)	.17
**Exposure characteristics**										
**Person to whom the employee was exposed was wearing a facemask at the time of exposure**										
Yes	566 (32.3)	77 (32.6)	489 (32.3)	Reference		38 (12.1)	7 (8.9)	31 (13.2)	Reference	
No	949 (54.2)	124 (52.5)	825 (54.5)	0.96 (0.74–1.25)	.77	244 (77.7)	68 (86.1)	176 (74.9)	1.51 (0.75–3.04)	.25
Not documented	235 (13.4)	35 (14.8)	200 (13.2)	1.09 (0.76–1.58)	.63	32 (10.2)	4 (5.1)	28 (11.9)	0.68 (0.22–2.11)	.50

Note. HCP, healthcare personnel; IQR, interquartile range; CI, confidence interval.

a Included employees who did not work in a patient care area.

b Included employees who worked in a patient care area in any capacity.

Reporting a high-risk exposure, whether occupational or community-based, as assigned by call-center operators, was significantly associated with a positive PCR test (Supplementary Table 1). Compared with employees with low-risk exposures, those with high-risk exposures had greater risk of a positive PCR test (RR, 1.39; 95% CI, 1.14–1.69). Employees with infected household contacts were at even greater risk of a positive PCR test (RR, 2.47; 95% CI, 2.11–2.89).

Throughout the study period, to conserve testing resources, employees who contacted the call center had to report at least 1 symptom concerning for COVID-19 to qualify for SARS-CoV-2 PCR testing, except in the context of cluster investigations, in which testing without symptoms was permitted. As a result, nearly all employees in our analysis (99%) reported at least 1 COVID-19 symptom. Among the 6,254 employees who reported ≥1 symptom (mean, 2 reported symptoms), 871 (13.9%) had a positive PCR test. Risk for a positive PCR test was highest among employees who reported loss of sense of smell (RR, 3.99; 95% CI, 3.51–4.53) or loss of sense of taste (RR, 3.35; 95% CI, 2.93–3.84) (Table 3). Cough, fever, muscle aches, joint aches, and congestion were also more frequently reported by employees with positive PCR tests, and sore throat, diarrhea, and nausea were more often reported by employees with negative PCR tests (Table 3). Employees who reported multiple symptoms were more likely to have a positive PCR test than employees reporting only 1 symptom (RR, 2.09; 95% CI, 1.62–2.70) (Table 3). In multivariable analysis, symptoms independently associated with positive SARS-CoV-2 PCR tests were cough, fever, muscle aches, loss of sense of taste, loss of sense of smell, and congestion (Table 4). Among employees reporting symptoms of COVID-19 during their call-center encounter, 40% stated that they had experienced symptoms while at work (Table 3), and 350 (40%) of these individuals went on to have a positive SARS-CoV-2 PCR test.

**Table 3.** Relative Risk of SARS-CoV-2 PCR Test Positivity Based on Reported Symptoms

Symptom	All Employees With Symptoms^a^ (N=6,254), No. (%)	Employees with a positive PCR Test (N=871), No. (%)	Employees With a Negative PCR Test (N=5,383), No. (%)	RR (95% CI)	*P* Value
**Reported symptoms**					
Sore throat	3,171 (50.7)	353 (40.5)	2,818 (52.3)	0.66 (0.58–0.75)	<.001
Cough	2,861 (45.8)	532 (61.1)	2,329 (43.3)	1.86 (1.64–2.11)	<.001
Fever	1,591 (25.4)	333 (38.2)	1,258 (23.4)	1.81 (1.60–2.05)	<.001
Muscle aches	1,771 (28.3)	329 (37.8)	1,442 (26.8)	1.54 (1.35–1.74)	<.001
Joint aches	903 (14.4)	173 (19.9)	730 (13.6)	1.47 (1.26–1.71)	<.001
Difficulty breathing	829 (13.3)	98 (11.2)	731 (13.6)	0.83 (0.68–1.01)	.063
Loss of sense of taste	413 (6.6)	167 (19.2)	246 (4.6)	3.35 (2.93–3.84)	<.001
Loss of sense of smell	386 (6.2)	181 (20.8)	205 (3.8)	3.99 (3.51–4.53)	<.001
Diarrhea	371 (5.9)	33 (3.8)	338 (6.3)	0.51 (0.36– 0.72)	<.001
Headaches^b^	1,522 (24.3)	227 (26.1)	1,295 (24.1)	1.10 (0.95–1.26)	.20
Fatigue^b^	1,020 (16.3)	136 (15.6)	884 (16.4)	0.95 (0.80–1.13)	.55
Congestion^b^	1,036 (16.6)	195 (22.4)	841 (15.6)	1.45 (1.26–1.68)	<.001
Nausea^b^	581 (9.3)	45 (5.2)	536 (10.0)	0.53 (0.40– 0.71)	<.001
Runny nose^b^	390 (6.2)	51 (5.9)	339 (6.3)	0.94 (0.72–1.22)	.62
Chest tightness^b^	150 (2.4)	17 (2.0)	133 (2.5)	0.81 (0.52–1.27)	.36
Reported >1 symptom	5,429 (86.8)	812 (93.2)	4,617 (85.8)	2.09 (1.62–2.70)	<.001
Symptoms while at work	2,482 (39.7)	350 (40.2)	2,132 (39.6)	1.02 (0.89–1.16)	.78
Not documented	465 (7.4)	63 (7.2)	402 (7.5)	0.98 (0.77–1.25)	.86

Note. RR, relative risk; CI, confidence interval.

a Employees had to report at least one symptom concerning for COVID-19 to qualify for SARS-CoV-2 PCR testing though the Call Center; however, some asymptomatic employees who were part of outbreak investigations also received testing.

b Symptom not specifically assessed via Call Center Script, but commonly reported as an “other” symptom.

**Table 4.** Symptoms Independently Associated with Positive SARS-CoV-2 PCR Test in Multivariable Analysis

Variable	RR (95% CI)^a^
Sore throat	0.64 (0.56–0.73)
Cough	1.95 (1.70–2.24)
Fever	1.79 (1.56–2.06)
Muscle aches	1.45 (1.26–1.66)
Difficulty breathing	0.70 (0.57– 0.87)
Loss of sense of taste	1.75 (1.40–2.20)
Loss of sense of smell	2.60 (2.09–3.24)
Congestion	1.29 (1.10–1.52)
Nausea	0.55 (0.40– 0.74)

Note. RR, relative risk; CI, confidence interval.

a Variables entered into model, but not retained: diarrhea, joint aches, reported more than one symptom. C-statistic, 0.74.

## Discussion

In this cohort of >6,000 health system and academic medical center employees early in the SARS-CoV-2 pandemic, a large proportion (52%) had no known SARS-CoV-2 exposure event. Among employees who reported a known exposure, community exposures, and particularly household exposures, were associated with greater risk of SARS-CoV-2 than occupational exposures. In addition, employees with higher-risk exposures, whether community or occupational, had greater risk of a positive PCR test than employees with low-risk exposures, and employees with infected household contacts were at greatest risk of a positive PCR test. This work highlights the importance of COVID-19 prevention in the community as well as the healthcare setting to prevent COVID-19 in HCP.^5–9^

The type of facility where employees worked was associated with risk for a positive PCR test. Employees working at the suburban community hospitals had greater risk of a positive PCR than those working at the urban academic hospital, whereas employees working in non-hospital settings had lower risk of a positive PCR. This observation may be related to differences in community rates of SARS-CoV-2 during the study period, to different thresholds for symptom reporting, or to differences in personal protective equipment (PPE) compliance at different facilities. The latter explanation deserves further investigation given that the association between working at a suburban community hospital and risk of a positive PCR test was evident for employees with occupational exposures but not for employees with community exposures.

HCP who provided direct patient care might be expected to be at greater risk to contract SARS-CoV-2 than other healthcare employees, and in our cohort, we observed that HCP who worked in patient care areas had greater risk for a positive PCR test than non-HCP who did not work in patient care areas. Previous studies that have compared SARS-CoV-2 infection rates among direct versus nondirect care providers have reported contradictory results: some reported higher rates of infection among direct care providers^5,10^ and others reported no difference^6,11^ or lower infection rates among direct care providers.^12^ Although further study is warranted, our findings suggest that HCP who have more contact with patients may have increased risk for contracting SARS-CoV-2.^13,14^ However, risk may depend on how frequently HCP care for COVID-19 patients and the types of PPE available and used. Although the facilities in our study had to use crisis and contingent PPE conservation strategies during the study period, including extended use or reuse of N95 masks, isolation masks, gowns, and face shields, they never ran out of PPE, and employees did not have to use cloth face coverings or nontraditional PPE, as reported in some other areas of the United States.^15,16^

Employees who reported community exposures were more likely to have positive PCR tests than those who reported occupational exposures. This finding emphasizes the importance of reducing risk of SARS-CoV-2 transmission from exposures that occur outside the hospital, as well as within the hospital. Several studies have reported that HCP are at increased risk of a positive SARS-CoV-2 PCR test as compared to others in the community.^17,18^ However, in studies in which a source of HCP infections was identified, most infections were attributed to community versus occupational exposures.^19,20^ Furthermore, studies that utilized genome sequencing to identify the source of SARS-CoV-2 infections in HCP have reported that most HCP infections were community acquired.^21^ However, other studies have attributed a high proportion of COVID-19 cases in HCP to occupational exposures,^22,23^ and it is likely that the actual risk of occupational transmission is dependent on exposure frequency, infection prevention precautions, and compliance with those precautions in the specific healthcare workplace.^24^

Throughout the COVID-19 pandemic, HCP have been concerned about the risk of contracting SARS-CoV-2 at work and bringing the virus home to their families. Although this remains an important concern, our data suggest that there should also be concern for the risk of SARS-CoV-2 transmission from an infected family member and bringing the virus to work. Moreover, 10% of the employees in our study reported having a household contact who was known or suspected to have COVID-19. Having an infected household contact more than doubled the risk of a positive PCR test. Other studies have also reported a high proportion of HCP that appear to have been infected within their household.^25,26^

One concerning finding of this study was that nearly 40% of employees reported having symptoms of COVID-19 while at work. Although this raises concerns about presenteeism, we were not able to determine whether these employees came to work while symptomatic or whether they developed symptoms while at work. Notably, our analysis only includes employees who chose to contact the call center, and symptomatic employees who chose not to self-report would not have been captured in our data. Therefore, actual rates of employees who were symptomatic while at work may be higher than reported. On the other hand, in a hypervigilant environment, employees with mild symptoms may have been encouraged to contact the call center about symptoms they would not have otherwise considered worrisome enough to report. Because other studies have reported a high rate of presenteeism among employees who report symptoms of COVID-19,^27^ policies and procedures that encourage employees to stay home from work when they are sick are needed.

Because the call center generally required employees to be symptomatic to be referred for SARS-CoV-2 testing during the study period, nearly all employees in our analysis reported symptoms concerning for COVID-19. The symptoms associated with positive SARS-CoV-2 PCR tests in our study (ie, loss of sense of smell and/or taste, fever, cough, muscle aches, and congestion) have been associated with positive PCR tests in other studies focusing on healthcare employees.^8,9,22,28–31^ Although shortness of breath and/or difficulty breathing is often considered a symptom of COVID-19,^22^ it was not associated with PCR positivity in our study. In our cohort, sore throat, diarrhea, and nausea were associated with lower risk for a positive SARS-CoV-2 PCR test. Although these symptoms are commonly associated with COVID-19, they may be less specific and could potentially be associated with other infectious diseases. A similar study of healthcare employees conducted in Massachusetts also reported that sore throat was associated with lower risk of a positive SARS-CoV-2 PCR test.^8^

This study is unique in that it includes detailed occupational, exposure, symptom, and SARS-CoV-2 PCR testing data from a large and diverse cohort of healthcare system and academic medical center employees. Our study had several limitations. We utilized data from a clinical database, including data that were primarily self-reported and could not be validated. Because testing was limited early in the pandemic, the call center was unable to offer PCR testing to asymptomatic individuals. As a result, most HCP in the study cohort were symptomatic. This selection bias likely led to a higher PCR positivity rate than would be expected if the cohort had included asymptomatic employees. Because the call-center script was updated frequently during the study period to account for emerging evidence regarding COVID-19 symptoms, prevention, and management, some data were not collected for all encounters. It was also difficult to determine which employees had direct patient contact based on the data collected, so some employees may have been incorrectly classified as HCP versus non-HCP. Although employees provided details about recent SARS-CoV-2 exposures, for those who had a positive SARS-CoV-2 PCR test, we could not be certain that the reported exposure caused their infection.

Despite these limitations, this study provides important data about SARS-CoV-2 among healthcare employees, including risk factors, symptoms of infection, and risks associated with occupational versus community exposures. Our findings indicate that community exposures, particularly household exposures, likely confer greater risk than occupational exposures for healthcare employees, including those with direct patient care. To reduce COVID-19 in HCP, measures to prevent COVID-19 in the community setting as well as the occupational setting are necessary.

## Supplementary material

For supplementary material accompanying this paper visit https://doi.org/10.1017/ash.2022.250. click here to view supplementary material
