# Supplementary material for: Clinical and occupational risk factors for coronavirus disease 2019 (COVID-19) in healthcare personnel
Source: Antimicrob Steward Healthc Epidemiol. 2022 Jul 18;2(1):e123. doi: 10.1017/ash.2022.250 (PMC9727206; doi:10.1017/ash.2022.250)
Supplement: Supplementary file 1 [file S2732494X22002509sup001.docx]

**Supplement Table 1**. Exposure Risk Assessment Categories used by the Call Center for asymptomatic employees with a COVID-19 exposure.

| **Exposure Category** | **Exposure** | **HCP Use of PPE during exposure** |
| --- | --- | --- |
| Low | PCC with a COVID-19 positive person who was wearing a facemask | Wearing a respirator, not wearing eye protection |
| Low | PCC with a COVID-19 positive person who was wearing a facemask | Wearing a respirator, not wearing gown or gloves |
| Low | PCC with a COVID-19 positive person who was wearing a facemask | Wearing all recommended PPE, but wearing a facemask instead of a respirator |
| Low | PCC with a COVID-19 positive person who was not wearing a facemask | Wearing a respirator, not wearing gown or gloves^ab^ |
| Low | PCC with a COVID-19 positive person who was not wearing a facemask | Wearing all recommended PPE, but wearing a facemask instead of a respirator |
| Medium | PCC with a COVID-19 positive person who was wearing a facemask | None |
| Medium | PCC with a COVID-19 positive person who was wearing a facemask | Not wearing a facemask or respirator |
| Medium | PCC with a COVID-19 positive person who was not wearing a facemask | Wearing a respirator, not wearing eye protection^a^ |
| High | PCC with a COVID-19 positive person who was not wearing a facemask | None |
| High | PCC with a COVID-19 positive person who was not wearing a facemask | Not wearing a facemask or respirator |
| Household Contact | Employee currently lives with someone who has tested positive or is awaiting COVID-19 testing results | Not applicable |
| Household Contact | Employee currently lives with someone who was not tested but is currently suffering from fever and respiratory illness | Not applicable |

Abbreviations: PPE=personal protective equipment; PCC = prolonged close contact (within 6 feet for >15 minutes over a 24 hour period)

^a^The risk category for these rows would be elevated by one level if the employee had extensive body contact with the patients (e.g., rolling the patient).

^b^The risk category for these rows would be elevated by one level if the employee performed or was present for a procedure likely to generate higher concentrations of respiratory secretions or aerosols (e.g., cardiopulmonary resuscitation, intubation, extubation, bronchoscopy, nebulizer therapy, sputum induction). For example, employees who were wearing a gown, gloves, eye protection and a facemask (instead of a respirator) during an aerosol-generating procedure would be considered to have a medium-risk exposure.
